# KRAS mutant rectal cancer cells interact with surrounding fibroblasts to deplete the extracellular matrix

**DOI:** 10.1002/1878-0261.12960

**Published:** 2021-06-15

**Authors:** Jin K. Kim, Michael R. Marco, Seo‐Hyun Choi, Xuan Qu, Chin‐Tung Chen, Moshe Elkabets, Lauren Fairchild, Oliver Chow, Francisco M. Barriga, Lukas E. Dow, Kevin O’Rourke, Bryan Szeglin, Dmitry Yarilin, Sho Fujisawa, Katia Manova‐Todorova, Philip B. Paty, Jinru Shia, Christina Leslie, J. Joshua Smith, Scott Lowe, Raphael Pelossof, Francisco Sanchez‐Vega, Julio Garcia‐Aguilar

**Affiliations:** ^1^ Department of Surgery Memorial Sloan Kettering Cancer Center New York NY USA; ^2^ Department of Epidemiology and Biostatistics Memorial Sloan Kettering Cancer Center New York NY USA; ^3^ Shraga Segal Department of Microbiology and Immunology The Cancer Research Centre Faculty of Health Sciences Ben‐Gurion University of the Negev Beer‐Sheva Israel; ^4^ Department of Computational and Systems Biology Memorial Sloan Kettering Cancer Center New York NY USA; ^5^ Department of Cancer Biology and Genetics Memorial Sloan Kettering Cancer Center New York NY USA; ^6^ Department of Medicine Weill‐Cornell Medical College New York NY USA; ^7^ Molecular Cytology Core Facility Memorial Sloan Kettering Cancer Center New York NY USA; ^8^ Department of Pathology Memorial Sloan Kettering Cancer Center New York NY USA; ^9^ Human Oncology and Pathogenesis Program Memorial Sloan Kettering Cancer Center New York NY USA; ^10^ Howard Hughes Medical Institute Memorial Sloan Kettering Cancer Center New York NY USA

**Keywords:** cancer‐associated fibroblast, extracellular matrix, KRAS, rectal cancer, tumor response, tumor stroma

## Abstract

Somatic mutations in the *KRAS* oncogene are associated with poor outcomes in locally advanced rectal cancer but the underlying biologic mechanisms are not fully understood. We profiled mRNA in 76 locally advanced rectal adenocarcinomas from patients that were enrolled in a prospective clinical trial and investigated differences in gene expression between *KRAS* mutant (KRAS‐mt) and *KRAS*‐wild‐type (KRAS‐wt) patients. We found that KRAS‐mt tumors display lower expression of genes related to the tumor stroma and remodeling of the extracellular matrix. We validated our findings using samples from The Cancer Genome Atlas (TCGA) and also by performing immunohistochemistry (IHC) and immunofluorescence (IF) in orthogonal cohorts. Using *in vitro* and *in vivo* models, we show that oncogenic *KRAS* signaling within the epithelial cancer cells modulates the activity of the surrounding fibroblasts in the tumor microenvironment.

## Graphical abstract

Oncogenic *KRAS* is associated with poor outcomes in locally advanced rectal cancer (LARC) but the underlying biologic mechanisms are not fully understood. We investigated differences in gene expression profiles between *KRAS* mutant and *KRAS* wild‐type LARC tumors. We found that *KRAS* mutant tumors display lower expression of genes related to the tumor stroma and remodeling of the extracellular matrix.

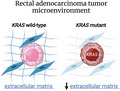

**Abbreviations**
CMS: consensus molecular subtypes
CRC: colorectal cancer
CRC‐IF cohort: colorectal cancer immunofluorescence cohort
CRC‐RT‐PCR cohort: colorectal cancer real‐time polymerase chain reaction cohort
CRT: chemoradiation
ECM: extracellular matrix
FFPE: formalin‐fixed paraffin‐embedded
GEMM: genetically engineered murine model
GSEA: gene set enrichment analysis
H&E: hematoxylin–eosin
IACUC: Institutional Animal Care And Use Committee
IF: immunofluorescence
IHC: immunohistochemistry
IRB: institutional review board
KRAS‐mt: *KRAS* mutant
KRAS‐wt: *KRAS*‐wild‐type
LARC: locally advanced rectal cancer
LARC‐IHC cohort: locally advanced rectal cancer immunohistochemistry cohort
LARC‐TCGA cohort: locally advanced rectal cancer‐the Cancer Genome Atlas cohort
LARC‐TIMING cohort: locally advanced rectal cancer‐Timing of Rectal Cancer Response to Chemoradiation trial cohort
MSI: microsatellite instability
MSK: Memorial Sloan Kettering
MSK‐IMPACT assay: Memorial Sloan Kettering‐Integrated Mutation Profiling of Actionable Cancer Targets assay
MSS: microsatellite stable
pCR: pathological complete response
RT‐PCR: real‐time polymerase chain reaction
ssGSEA: single‐sample gene set enrichment analysis
TCGA: the Cancer Genome Atlas
TIMING trial: Timing of Rectal Cancer Response to Chemoradiation trial

## 1 Introduction

Colorectal cancer (CRC) is the third most common cancer diagnosed in the United States, with almost 150 000 estimated cases and more than 50 000 expected deaths in 2021 [1]. *KRAS* mutations are detected in ~ 40% of all colorectal cancers [2, 3, 4] and they are associated with worse prognosis and more aggressive tumor behavior [5, 6]. While CRC has been historically considered a uniform disease, recent evidence increasingly suggests that anatomical location is associated with important differences in epidemiological, molecular, and clinical features [4, 7]. Within the setting of metastatic CRC, *KRAS* mutations have been associated with treatment resistance [8] as well as worse survival after liver metastasectomy [9]. In locally advanced rectal cancer (LARC), *KRAS* mutations have been identified as a biomarker of poor response to neoadjuvant chemoradiation (CRT), with *KRAS* and *TP53* double mutants exhibiting particularly low rates of response [10, 11, 12]. While the resistance of KRAS‐mt CRC to anti‐EGFR therapy can be explained by the constitutive activation of the MAPK pathway [13], the mechanisms of resistance to nontargeted therapies (such as CRT) remain largely unexplored.

Prior transcriptomic studies have failed to identify reliable predictive features of response likely due to a multitude of factors, including small sample sizes, heterogeneous patient cohorts, and nonuniform definitions of tumor response [14, 15]. In this study, we present a molecular analysis of LARC tumors from patients accrued through the Timing of Rectal Cancer Response to Chemoradiation (TIMING) clinical trial [16]. This is a phase 2, nonrandomized, multicenter clinical trial that evaluated the proportion of LARC patients achieving a pathological complete response (pCR) after being treated with neoadjuvant CRT followed by various doses of consolidative chemotherapy. This unique cohort provides an excellent opportunity to analyze a clinically homogenous population of patients that were selected and treated within the context of a clinical trial and that were evaluated based on uniform assessments of response. Since *KRAS* mutational status has been previously reported as a strong biomarker of response in this cohort [10], we sequenced mRNA for a subset of 76 patients and investigated differences in the transcriptomic profile of KRAS‐mt and KRAS‐wt tumors in order to gain insights into the mechanisms by which *KRAS* mutations increase tumor aggressiveness.

We identified dysregulation of the extracellular matrix (ECM) as the strongest discriminating feature between KRAS‐mt and KRAS‐wt LARC. The tumor stroma is composed of fibroblasts and the ECM, a three‐dimensional arrangement of molecules that provide biomechanical and biochemical support to the surrounding cells [17]. Accumulating evidence suggests that the tumor stroma plays a crucial role in cancer progression and treatment resistance [18, 19, 20]. Using genetically engineered murine models (GEMM) and tumor‐fibroblast co‐cultures, we show that KRAS‐mt epithelial cancer cells downregulate the expression of ECM‐related genes in the neighboring fibroblasts.

## 2 Materials and methods

### 2.1 Sample inclusion criteria

Our main cohort (LARC‐TIMING cohort) consisted of 76 LARC pretreatment biopsy samples from patients accrued through the TIMING trial [16] (ClinicalTrials.gov, number NCT00335816). Local staging was performed by endorectal ultrasound or magnetic resonance imaging and patients were screened for metastatic disease with computed tomography scans. These tumors were profiled for transcriptomic analyses using gene expression microarrays, and the relevant clinicopathological information is provided as Table S1. All these patients had American Joint Committee on Cancer clinical stage II (T3‐4, N0) or III (any T, N1‐2) rectal adenocarcinoma with a distal tumor border within 12 cm of the anal verge. Inclusion was contingent on an adequate amount of tissue to allow for molecular analysis. Tumors with microsatellite instability (MSI) were excluded from our analysis because they comprise less than 5% of rectal cancer and present very different biologic and clinical features [2, 21, 22]. The study methodologies conformed to the standards set by the Declaration of Helsinki. Consent to analyze specimens from patients was obtained after approval by a central Institutional Review Board (IRB) at Memorial Sloan Kettering (MSK) and the individual IRBs at the participating institutions. Gene expression data is uploaded in GEO: (GSE170999, https://www.ncbi.nlm.nih.gov/geo/query/acc.cgi?acc=GSE170999).

We demonstrate the reproducibility of our findings at the transcriptional level using an external validation cohort of 71 microsatellite stable (MSS) LARC tumors from The Cancer Genome Atlas (LARC‐TCGA cohort), for which RNA‐Seq data are publicly available [23]. The final set of TCGA barcodes used in our analyses, together with relevant clinical features, is provided as Table S2. To further validate some of our results, we performed IHC in a set of 23 LARC pretreatment biopsy specimens (LARC‐IHC cohort) from patients treated at MSK. RNA‐seq was performed for validation in 15 of these 23 patients with usable biological material at the time. Due to the limited availability of pretreatment rectal cancer tissue, technical validation of some of our findings was also done on stage I‐III colon adenocarcinoma tissues from patients that underwent upfront resection at MSK. This included a set of 35 colon adenocarcinoma resection specimens that were used for immunofluorescence staining (CRC‐IF cohort). We also used real‐time polymerase chain reaction (RT‐PCR) in a set of 45 colon adenocarcinomas to validate the expression of select genes (CRC‐RT‐PCR cohort). Consent to analyze specimens from patients treated at MSK was obtained after approval by our IRB.

### 2.2 DNA and RNA extraction from tissue blocks

Pretreatment biopsies were obtained from patients in the LARC‐TIMING cohort and patients in the LARC‐IHC cohort at the time of diagnosis. All the specimens were stored as formalin‐fixed paraffin‐embedded (FFPE) blocks and ten slides of 10‐micron thickness were cut per block. A pathologist reviewed the hematoxylin and eosin (H&E) slides of all the sections to confirm the boundaries of the malignant epithelia. The tumor‐enriched areas were macrodissected from unstained slides guided by the H&E slides. DNA & RNA were extracted from FFPE sections using AllPrep DNA/RNA FFPE kit (Qiagen Inc., Valencia, CA, USA).

### 2.3 RNA profiling

Two separate platforms (Affymetrix microarray and Hiseq) were used to analyze the LARC‐TIMING cohort and the LARC‐IHC cohort, respectively. For the Affymetrix platform, total RNA was amplified to generate cDNA libraries using the Ovation FFPE WTA System (NuGEN Technologies, San Carlos, CA, USA) and sent for Affymetrix U133 Plus 2.0 Array (Affymetrix, Santa Clara, CA, USA). For the Hiseq platform, total RNA was used to generate libraries for mRNA deep sequencing using an adapted version of the Illumina v1.5 protocol and optimizing for reaction volume. Sequencing was done using the Illumina HiSeq 2000 Platform (Illumina, San Diego, CA, USA).

### 2.4 DNA sequencing and mutation calling

Two platforms were used for DNA sequencing. For the LARC‐TIMING cohort, *KRAS* mutations were determined by standard PCR followed by Sanger sequencing of exons 2 and 3 of *KRAS*. MSI status was tested by PCR for the five‐marker panel (BAT‐25, BAT‐26, MONO‐27, NR‐21, and NR‐24) using Promega’s MSI Analysis System v1.2 (Promega, Madison, WI, USA). LARC‐IHC cohort specimens and CRC‐IF cohort specimens were analyzed using Memorial Sloan Kettering‐Integrated Mutation Profiling of Actionable Cancer Targets (MSK‐IMPACT) assay to generate somatic mutational profiles from common oncogenes as published previously [24]. In select cases where the *KRAS* mutation calls were discordant between Sanger sequencing and MSK‐IMPACT, we elected to follow the calls made by MSK‐IMPACT. MSI status for the both LARC‐IHC and CRC‐IF cohorts was assessed using scores computed using MSISensor [25] with a cut‐off value of 10.

### 2.5 Computational analyses

Computational analyses were performed in the ‘R’ statistical environment, version 3.6.0. A background correction using the robust multiarray average method was performed on the microarray data. Low count genes with an aggregate count below 50 across all samples were filtered out. Probes without matching Entrez Gene IDs and probes that map to multiple gene symbols were removed. Data normalization was conducted using conditional quantile normalization from the R package cqn. Differential analysis of mRNA expression was conducted using the ‘DESeq2’ package in R [26]. Significant differences were required to exhibit |log_2_FoldChange| > 1 and FDR < 0.05. Gene set enrichment analysis (GSEA) was conducted using median expression values across probes associated with each gene with the ‘fgseaMultilevel’ function from the ‘fgsea’ package in R, which is based on the adaptive multilevel splitting Monte Carlo approach. Prerank scores were calculated as –log_10_(*P*‐value)* sign(log_2_FoldChange). The following packages were utilized in the analysis: BiocManager 1.30.9, DESeq2 1.24.0, biomaRt 2.41.6, fgsea 1.12.0, GSVA 1.34.0, genefilter 1.68.0, GSEABase 1.48.0, Biobase 2.46.0, cqn 1.32.0, CancerSubtypes 1.12.1, CMScaller 0.99.1, EnhancedVolcano 1.4.0, ggplot2 3.3.0, enrichplot 1.6.1, ggpubr 0.3.0, ComplexHeatmap 2.2.0, grDevices 3.6.3, affy 1.64.0, TCGAbiolinks 2.14.1. Single‐sample gene set enrichment analysis (ssGSEA) was performed using the R package ‘GSVA’. For the RT‐PCR plot in Fig. S2A, fold change was calculated after normalization to human 18s rRNA using the $2^{‐\Delta \Delta C_{t}}$ method; one‐sided Wilcoxon rank‐sum test was then performed. For IHC and IF, the median value per slide was taken and a one‐sided Wilcoxon rank‐sum test was performed. For the RT‐PCR plots in Fig. 4, each experiment was done in biological and technical triplicate and fold change of gene expression was calculated after normalization to human GAPDH using the $2^{‐\Delta \Delta C_{t}}$ method; one‐sided *t*‐test was performed.

### 2.6 Tissue staining and protein quantification

Sequential 5‐micron‐thick slides were prepared for H&E and IF or IHC staining. IF staining was done for DAPI, VIM, FN, and POSTN by the MSK Molecular Cytology Core. IHC staining was done for POSTN. Table S8 contains the list of antibodies used. All slides were digitally scanned with Panoramic Flash (3DHistech, Budapest, Hungary) using 20×/0.8NA objective and appropriate filters. Scanned data were reviewed by a pathologist who was blinded to the molecular data. Relevant regions of tumor core with adjacent stroma were annotated using CaseViewer (3DHistech) with H&E as a guide. Annotated regions were quantified using custom macros written in fiji/imagej (NIH, Bethesda, MD, USA). For IHC POSTN signal, DAB and hematoxylin staining were separated using the Color Deconvolution algorithm and areas of threshold positive signal were measured and normalized to the area of tissue. For IF‐stained signals, the number of total cells and positive cells was tabulated. The median value per slide was taken, and a Wilcoxon rank‐sum test was performed for statistical analysis.

### 2.7 Genetically engineered mouse models

All experiments were performed in accordance with the Memorial Sloan Kettering Institutional Animal Care and Use Committee (IACUC) under protocol number 11‐06‐012. Doxycycline was administered via food pellets (625 mg·kg^−1^) (Harlan Teklad). 4‐Hydroxytamoxifen (4OHT, Sigma Aldrich, St. Louis, MO, USA, 70% Z‐isomer) was delivered by a single intraperitoneal injection (0.5 mg/mouse) at 5–6 weeks of age. LSL‐Kras (B6.129S4‐Kras^tm4Tyj^/J) and Lgr5‐CreER (B6.129P2‐*Lgr5^tm1(cre/ERT2)Cle^*/J) animals were purchased from Jackson Laboratories. CAGs‐LSL‐rtTA3 (B6. Cg‐*Gt(ROSA)26Sor^tm1(CAG‐rtTA3)Slowe^*/LdowJ) and TG‐Apc.3374 (Col1a1^tm4(tetO‐GFP/RNAi:Apc)Slowe^) mice were described previously [27, 28]. We used a GEMM in which the *Apc* gene can be conditionally suppressed using a doxycycline‐regulated shRNA to develop colon tumors (*shApc/Kras^wt^* mice), as described previously [27, 28]. A KRAS‐mt line was developed by crossing conditional KRAS‐mt allele‐carrying mice (*LSL‐Kras^G12D^*) with the *shApc* mice to develop *shApc/Kras^G12D^* mice. Mouse colonic organoids were isolated as previously published [28].

### 2.8 Co‐culture of human colorectal cancer cell lines and mouse fibroblasts

Caco2 cells, a MSS colorectal cancer cell line, with or without *KRAS^G12V^* mutation [29] were cultured in MEM supplemented with 20% FCS, 1× nonessential amino acids, 2 mm l‐glutamine, and penicillin–streptomycin. Mouse fibroblast NIH‐3T3 (ATCC, Manassas, VA, USA, CRL‐1658) was cultured in Dulbecco's Modified Eagle's medium (DMEM) supplemented with 10% FCS, 4 mm l‐glutamine, 1 mm sodium pyruvate, and penicillin–streptomycin. NIH‐3T3 culture media was used in co‐culture experiments. For direct co‐culture, NIH‐3T3 cells (2 × 10^5^ cells) were plated into 100‐mm dish with Caco2 cells (2 × 10^5^ cells). For Transwell co‐culture, NIH‐3T3 cells (1 × 10^5^ cells) and Caco2 cells (1 × 10^5^ cells) were plated into 0.4 mm pore sized 6‐well Transwell inserts (Corning, Glendale, AZ, USA, Cat#353493) and the lower chamber, respectively.

### 2.9 Co‐culture of mouse colonic organoids and human fibroblasts

*shApc/Kras^wt^* or *shApc/Kras^G12D^* mouse colonic organoids were cultured in DMEM/F12 supplemented with 25% Wnt3a conditioned media, 50 ng·mL^−1^ recombinant mouse EGF (Invitrogen, Carlsbad, CA, USA, PMG8043), 10% Noggin conditioned media, 10% R‐spondin conditioned media, 1 mm N‐acetyl cysteine (Sigma, A9165), 10 mm nicotinamide (Sigma, N0636), 10 mm HEPES pH 7.3 (Quality Biological, Gaithersburg, MD, USA, 118‐089‐721), 1× penicillin–streptomycin (GIBCO, 15140‐122) 0.5 μg·mL^−1^ doxycycline (Sigma, D9891) in Advanced DMEM/F12 (GIBCO, 12634‐010). *Apc* ^−/−^ *Trp53* ^−/−^ *Kras^wt^* and *Apc* ^−/−^ *Trp53* ^−/−^ *Kras^G12D^* mouse colonic organoids were derived from *Apc^flox/flox^*; *Trp53^flox/flox^* and *Apc^flox/flox^*;LSL‐*Kras^G12D/+^*; *Trp53^flox/flox^* mice. Freshly isolated crypts were mixed with adenoviral Cre (Vector Biolabs, Malvern, PA, USA, 1045) and embedded in Matrigel for loxP site recombination. Recombined organoids were selected and cultured in the above media without Wnt3a, R‐spondin, or doxycycline. Human normal colonic fibroblast cell line CCD‐18Co (ATCC, CRL‐1459) was cultured in DMEM supplemented with 10% FCS, 1× nonessential amino acids, 2 mm l‐glutamine, and penicillin–streptomycin.

For direct co‐culture, the mouse colonic organoids were released from Matrigel (Corning, 356231), using Cell Recovery Solution (Corning, 354253), dissociated using TrypLE TM Express (Invitrogen, 12605028), and filtered through 30‐μm nylon mesh (Miltenyi, Bergisch Gladbach, North Rhine‐Westphalia, Germany, 130‐041‐407). The dissociated organoids (5 or 7.5 × 10*^3^* cells) were suspended with human fibroblasts (5 × 10*^4^* cells) in 50 μL Matrigel and plated in a 24‐well plate (Greiner Bio‐One, St. Louis, MO, USA, 662‐102).

For Transwell co‐culture, the human fibroblasts (2 × 10*^5^* cells) were plated into 0.4‐mm pore sized 6‐well Transwell inserts (Corning, Cat#353493) and the dissociated organoids (3 × 10*^4^* cells or 2 × 10*^4^* cells) in 200 µL Matrigel were plated in the lower chamber (Greiner Bio‐One, 657‐185). Culture media prepared for *shApc/Kras* was used for its co‐culture. A 3 : 1 mixture of organoid culture media and human fibroblast culture media was used for co‐culture with CCD‐18co + *Apc* ^−/−^ *Trp53* ^−/−^ *Kras*. Cells were harvested after 4–5 days for gene expression analysis.

### 2.10 RNA isolation from co‐cultures and RT‐PCR

Cells were released using Cell Recovery Solution and pellets harvested for RNA isolation. Total RNA was isolated using TRIzol reagent (Ambion, Austin, TX, USA, 15596018) and RNeasy Mini kit (Qiagen, 74104), and cDNA was synthesized using a TaqMan Reverse Transcription Reagents (Applied Biosystems, Foster City, CA, USA, N8080234). All targets were amplified using TaqMan 2× Universal PCR master mix (Applied Biosystems, 4304437) and gene‐specific Taqman primers and probe sets (Applied Biosystems) in ViiA 7 real‐time PCR system (Applied Biosystems). Primers used are listed in Table S7. Fold change of gene expression was calculated after normalization to human GAPDH, using the $2^{‐\Delta \Delta C_{t}}$ method.

### 2.11 RT‐PCR analysis from frozen tissue

Total RNA was extracted from frozen tissue specimens of the CRC‐RT‐PCR cohort using TRIzol reagent (Ambion, 15596018) and Qiagen’s RNeasy Mini kit (Qiagen Inc.). cDNA was synthesized using a TaqMan Reverse Transcription Reagents (Applied Biosystems, N8080234). All targets were amplified using TaqMan 2× Universal PCR master mix (Applied Biosystems, 4304437) and gene‐specific Taqman primers and probe sets (Applied Biosystems) in ViiA 7 real‐time PCR system (Applied Biosystems). Primers used are listed in Table S7.

## 3 Results

### 3.1 Study Design and Cohort Description

**Fig. 1:**
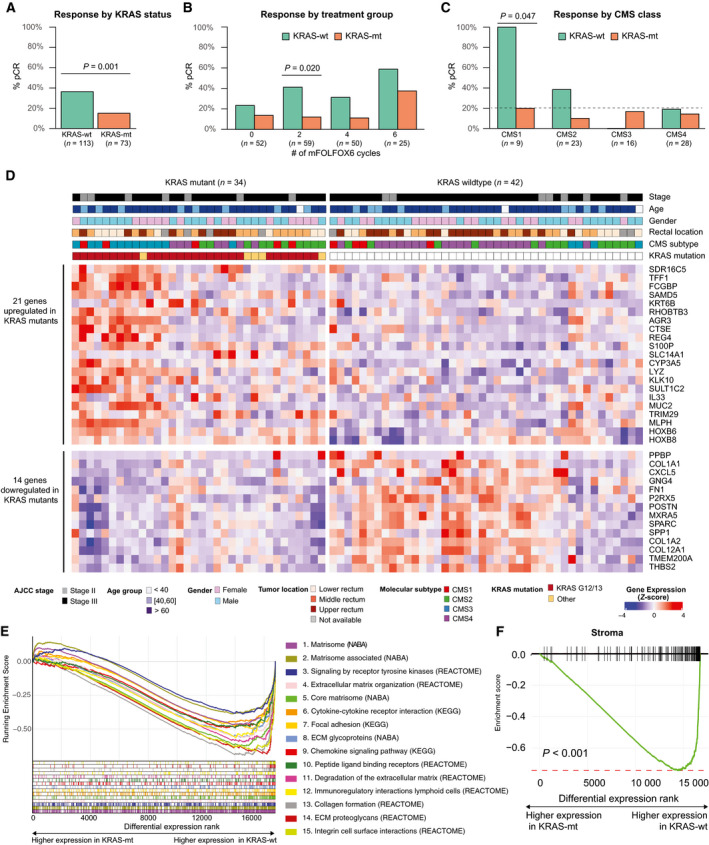
*KRAS* mutant LARC tumors have a distinct transcriptomic signature notable for dysregulated stromal genes and genes coding ECM‐related proteins. (A) *KRAS* mutations are associated with lower fractions of complete pathological response in 186 sequenced patients from the TIMING trial (*P* = 0.001). (B) KRAS‐mt patients have consistently lower fractions of pCR across all treatment groups. pCR rates were significantly different by *KRAS* status in the treatment subgroup with the greatest number of patients (*n* = 59, *P* = 0.020). All patients received chemoradiation (fluorouracil 225 mg·m^−2^ per day by continuous infusion throughout radiotherapy, and 45 Gy in 25 fractions, 5 days per week for 5 weeks, followed by a minimum boost of 5·4 Gy). Each cycle of mFOLFOX6 consisted of racemic leucovorin 200 or 400 mg·m^−2^, oxaliplatin 85 mg·m^−2^ in a 2‐h infusion, bolus fluorouracil 400 mg·m^−2^ on day 1, and a 46‐h infusion of fluorouracil 2400 mg·m^−2^. (C) Based on mRNA data for 76 patients, KRAS‐mt tumors also exhibited consistently lower fraction of pCR across all molecular subtypes. pCR rates by *KRAS* status were significantly different in CMS1 (*P* = 0.047). Only statistically significant changes based on Fisher’s test are marked in figures (A‐C). (D) Heatmap of differentially expressed genes (FDR<0.05, |log_2_FoldChange| > 1) in KRAS‐mt and KRAS‐wt LARC pretreatment tumors from the LARC‐TIMING cohort. (E) Gene set enrichment analysis (GSEA) enrichment plots for the 15 most significant pathways in our cohort, ranked by statistical significance (Table S5), reveal significant downregulation of ECM and matrisome‐related genes in KRAS‐mt tumors. (F) GSEA enrichment plot using the ESTIMATE stromal signature shows significant downregulation of stroma‐related genes in KRAS‐mt tumors.

*KRAS* mutational status was available for 186 LARC patient samples from the TIMING trial that had been profiled using a combination of Sanger sequencing and next‐generation targeted sequencing, as previously described [10]. *KRAS* mutations were identified in 73/186, 39.2% of patients. Patients with KRAS‐mt tumors exhibited significantly lower rates of pCR than patients with KRAS‐wt tumors (15% vs 36%, *P* = 0.0015) (Fig. 1A). KRAS‐mt patients also had consistently lower response rates across all four neoadjuvant treatment groups (Fig. 1B).

We profiled mRNA for 76 of these tumors using the Affymetrix U133 platform (LARC‐TIMING cohort). There were no significant differences in the clinicopathological characteristics of the profiled KRAS‐mt and KRAS‐wt tumors (Table 1 and Table S1). Detailed examination of the transcriptomic data based on the consensus molecular subtypes (CMS) classifier [21] revealed similar patterns to those that have been widely reported in colorectal cancer; notably, overexpression of immune‐related genes in CMS1 and genes related to epithelial–mesenchymal transition in CMS4 (Fig. S1). Missense mutations in *KRAS* with known functional relevance were observed in 34/76 (44%) of the patients. Most of these mutations occurred in codons G12 (23/34, 67%) and G13 (7/34, 20%). KRAS‐mt tumors were enriched in CMS3 (12/16, 75% vs 22/60, 36%; *P* = 0.002), consistent with prior reports [21]. When looking at tumor response by CMS, we found that the response rates varied by CMS group with CMS1 displaying the highest response rate (55%) whereas CMS3 displayed the lowest response rate (12.5%). KRAS‐mt tumors exhibited consistently worse response rates than KRAS‐wt tumors across all CMS groups (Fig. 1C).

**Table 1:** Clinicopathological Characteristics of the LARC‐TIMING Cohort. Data *n* (%) or mean (stdev). *P*‐value by Fisher’s exact test, *t*‐test.

	KRAS‐mt (*n* = 34)	KRAS‐wt (*n* = 42)	*P*‐value
Age (years)	56.9 (11.7)	55.6 (10.7)	0.6
Male gender	19 (56%)	24 (57%)	> 0.9
Tumor distance from anal verge (cm)	6.5 (3.3)	7.0 (3.4)	0.5
cT classification			
T2	5 (15%)	4 (9%)	0.36
T3	29 (85%)	36 (86%)	
T4	0 (0%)	2 (5%)	
cN classification			
0	7 (20%)	8 (19%)	> 0.9
1	23 (68%)	29 (69%)	
2	4 (12%)	5 (12%)	
Clinical stage			
II	7 (21%)	8 (19%)	> 0.9
III	27 (79%)	34 (81%)	
MSS	34 (100%)	24 (100%)	
Treatment group			
CRT+ 2 cycles of mFOLFOX	18 (53%)	13 (31%)	0.064
CRT+ 4 cycles of mFOLFOX	16 (47%)	29 (69%)	
Complete responder	5 (15%)	13 (31%)	0.11
CMS subtype			
CMS1	5 (15%)	4 (10%)	0.013
CMS2	10 (29%)	13 (31%)	
CMS3	12 (35%)	4 (10%)	
CMS4	7 (21%)	21 (50%)	

### 3.2 *KRAS* mutant LARC tumors exhibit a distinct transcriptomic profile

To gain further insight into the biological role of *KRAS* mutations in LARC, we investigated differences in gene expression between KRAS‐mt and KRAS‐wt tumors. A set of 35 genes were differentially expressed, including 21 upregulated genes and 14 downregulated genes in KRAS‐mt specimens (Fig. 1D and Table S3). We demonstrated the reproducibility of our results by using RT‐PCR to quantify the expression of two significantly upregulated genes (*HOXB6*, *P* = 0.02; *HOXB8*; *P* = 0.01) and two significantly downregulated genes (*COL1A1*, *P* = 0.04; *SPARC*, *P* = 0.03) in an independent set of colon adenocarcinomas (CRC‐​RT‐PCR cohort) composed of 30 KRAS‐wt and 15 KRAS‐mt tumors (Fig. S2A). The differential gene expression between KRAS‐mt and KRAS‐wt MSS LARC was also validated using RNA‐Seq from an independent set of 71 LARC specimens from TCGA (LARC‐TCGA cohort) [2, 23]. Both the set of upregulated and downregulated genes that we had originally identified were also differentially expressed (*P* = 0.001 and *P* < 0.001, respectively) in the LARC‐TCGA cohort (Fig. S2B, Table S4).

### 3.3 *KRAS* mutations in LARC are associated with changes in the stroma

Genes upregulated in KRAS‐mt tumors included *HOXB6* and *HOXB8*, two homeobox genes that have been previously reported to be dysregulated in colorectal cancer [30]. We observed increased levels of *S100P*, which encodes a member of the S100 calcium‐binding proteins, and whose upregulation has been linked to increased metastatic potential and decreased chemosensitivity in colorectal cancer [31]. KRAS‐mt tumors also exhibited higher expression levels of interleukin 33 (*IL‐33*), a regulator of stromal cell activation that has been reported to contribute to the formation of a protumorigenic microenvironment [32]. We performed GSEA [33] using the Canonical Pathways collection from the Molecular Signatures Database (MSigDB) from the Broad Institute [34] and found that ECM remodeling and matrisome‐related biological processes [35] clearly stood out as the top pathways being differentially regulated in KRAS‐mt vs KRAS‐wt tumors (Fig. 1E, Table S5). In particular, the set of genes downregulated in KRAS‐mt tumors was strongly enriched in genes encoding ECM remodeling proteins such as periostin (*POSTN*), fibronectin I (*FN1*), thrombospondin 2 (*THBS2*), osteopontin (*SPP1*), osteonectin (*SPARC*), adlican (*MXRA5*), and several collagen alpha chains (*COL1A1, COL1A2, COL12A1)* (Fig. 1D and Tables S3,S5). As the ECM is mainly regulated by fibroblasts, we postulated that changes in the stromal cells of the tumor microenvironment may be responsible for this pronounced dysregulation of ECM‐related genes. We evaluated this hypothesis using the previously published ESTIMATE gene signature for stromal infiltration [19], and we found significantly reduced stromal gene expression in KRAS‐mt compared to KRAS‐wt tumors (Fig. 1F). Furthermore, our GSEA results showing significant differences in the ECM‐related pathways and stromal signature were validated using the LARC‐TCGA cohort (Fig. S2C,D, Table S6).

**Fig. 2:**
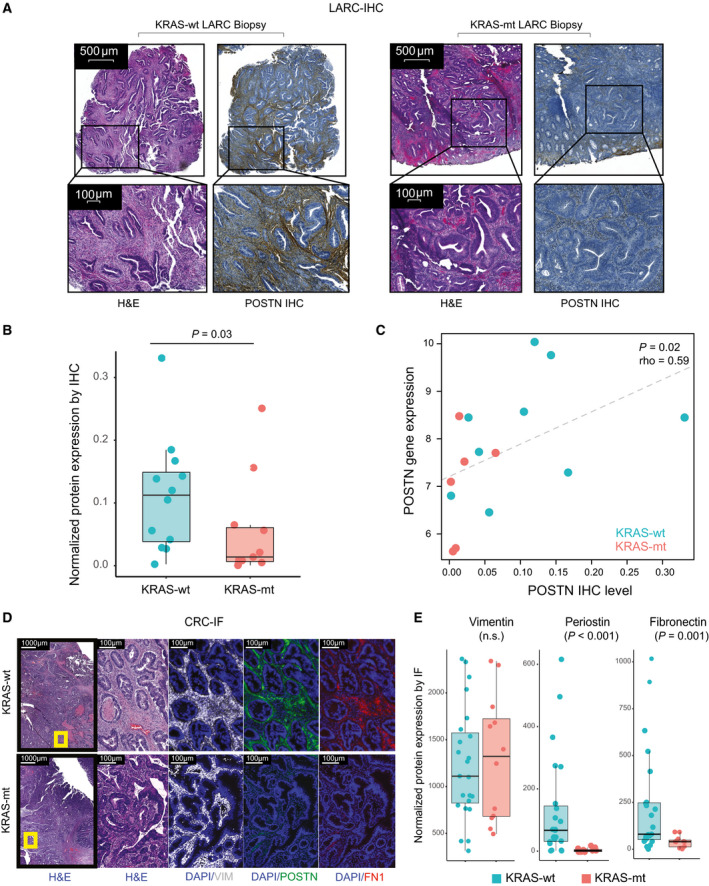
Expression of ECM proteins in the tumor stroma is reduced in KRAS‐mt CRC. (A) Representative images of POSTN IHC staining in KRAS‐mt (*n* = 11) vs KRAS‐wt (*n* = 12) LARC biopsies. Scale bar corresponds to 500 μm for the upper panels and 100 μm for the lower panels. (B) Quantification of POSTN IHC staining in KRAS‐mt (*n* = 11) vs KRAS‐wt (*n* = 12) LARC biopsies. KRAS‐mt tumors have lower POSTN levels than KRAS‐wt tumors (*P* = 0.03). Median value from 3 to 5 quantified tumor areas per sample (threshold intensity/mm2) is plotted. (C) RNA expression of *POSTN* via RNAseq correlates with protein expression (assessed with Spearman’s correlation, *P* = 0.02, rho=0.59); *n* = 9 KRAS‐wt, *n* = 6 KRAS‐mt. R^2^=0.22 for the linear model approximation. (D) IF of VIM, POSTN, FN1 in KRAS‐mt (*n* = 12) vs KRAS‐wt (*n* = 23) CRC specimens. Scale bar corresponds to 1000 μm on the left most panels and 100 μm on the other panels. (E) Levels of VIM, POSTN, and FN1 expression on IF. Median value from 6 quantified tumor areas per sample (intensity/mm2) is plotted. POSTN and FN1 expression is lower in KRAS‐mt versus KRAS‐wt tumors (*P* < 0.001 and *P* = 0.001, respectively). Whiskers in B, E box plots represent 1.5× the interquartile range. *P*‐values in B, E were computed using one‐sided Wilcoxon rank‐sum tests.

We then employed IHC for orthogonal validation of our results by quantifying differences in the protein expression of POSTN, a key regulator of collagen cross‐linking of the ECM, which serves as a ligand for alpha‐V/beta‐3 and alpha‐V/beta‐5 integrins to support adhesion and migration of epithelial cells [36]. In normal colorectal tissue, POSTN is expressed primarily in the lamina propria surrounding the crypts, but not in the epithelial cells or in the submucosa [36] (Fig. S3). We stained MSS LARC biopsies (*n* = 12 KRAS‐wt; *n* = 11 KRAS‐mt), which were obtained from an independent patient cohort. We found that POSTN staining intensity was significantly lower in KRAS‐mt compared to KRAS‐wt tumors (*P* = 0.03, Fig. 2A,B). We performed RNA‐Seq in a subset of 15 cases (*n* = 9 KRAS‐wt; *n* = 6 KRAS‐mt), and we found that the IHC staining intensities were significantly correlated with their associated levels of mRNA expression (*P* = 0.02, Fig. 2C).

To further analyze changes in the stromal composition of KRAS‐mt vs KRAS‐wt tumors, we used transcriptomic signatures for a set of 14 distinct stromal cell populations that had been identified by performing single‐cell RNA sequencing of 5993 cells from 23 colorectal cancer patients in a recently published study [37]. Consistent with the results observed in our LARC‐TIMING cohort, we found that the gene expression of a majority of these stromal cell types was depleted in KRAS‐mt tumors (Fig. S4A,B). In particular, differences were driven by reduced transcriptional activity of fibroblasts, including both myofibroblast‐specific signatures and signatures for other stromal fibroblasts. By contrast, transcriptional activity for diverse subtypes of endothelial cells showed no significant differences or was actually increased in the KRAS‐mt samples. Among the myofibroblast‐specific signatures, the strongest decrease was observed in type 1 and 2 myofibroblasts, which were characterized by genes involved in ECM synthesis and degradation, respectively. Of note, no significant differences were observed in the levels of type 3 myofibroblasts, which were characterized by *ACTA2* and *RGS5* expression and therefore thought to have a pericyte origin.

### 3.4 Decreased expression of ECM‐related genes in KRAS‐mt tumors is not explained by differences in tumor cellularity

To resolve whether or not the changes in the stromal gene signature expression were simply due to differences in the fraction of stromal versus cancer cells in KRAS‐mt vs KRAS‐wt tumors, we performed multiplex IF staining on KRAS‐wt (*n* = 23) and KRAS‐mt (*n* = 12) MSS colon cancer specimens (Fig. 2D,E). Levels of vimentin (VIM), an intermediate filament protein expressed by fibroblasts, were not different in KRAS‐mt and KRAS‐wt tumors, demonstrating that the number of fibroblasts was comparable. This is consistent with the lack of significant differences in *VIM* mRNA expression between KRAS‐mt and KRAS‐wt tumors in our LARC‐TIMING cohort (*q* = 0.2). By contrast, *POSTN* and *FN1*, which are two ECM‐associated genes that were differentially expressed in our *KRAS* signature, had diminished protein expression in KRAS‐mt compared to KRAS‐wt specimens (Fig. 2D,E). We also analyzed computational estimates of tumor purity (defined as the ratio of cancer vs noncancer cells in the sequenced samples) for the LARC‐TCGA cohort that had been obtained using the ABSOLUTE algorithm based on SNP6 array data from TCGA [38], and no significant differences were observed between KRAS‐mt and KRAS‐wt specimens (Fig. S2E). These results are all consistent with the observed transcriptional signature being predominantly related to a fibroblast cell state change rather than cellularity effects.

### 3.5 Mutational activation of *KRAS* induces the observed stromal signature

**Fig. 3:**
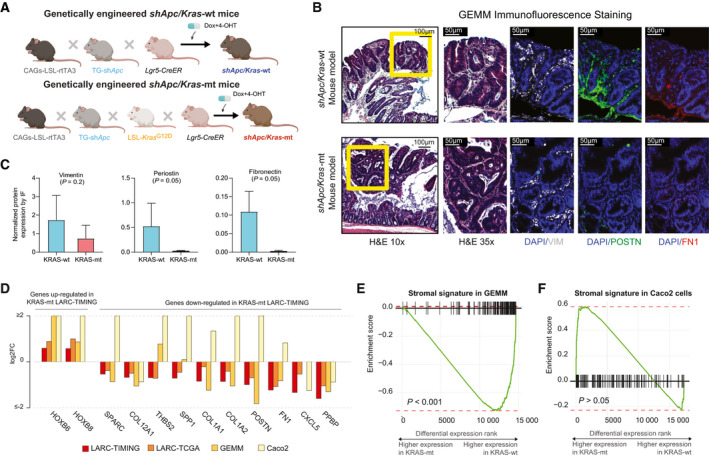
Expression of ECM‐related proteins by cancer‐associated fibroblasts is reduced in KRAS‐mt tumors. (A) Transgenic mice with reverse tet‐transactivator (rtTA3) in a LSL cassette were crossed with TRE‐regulated, GFP‐linked short‐hairpin Apc (TG‐sh*Apc*) and then crossed with *Lgr5*‐hydroxytamoxifen (4‐OHT) inducible Cre (*Lgr5*‐CreER). These mice were then treated with doxycycline (Dox) and 4‐OHT to generate sh*Apc/Kras*‐wt mice. The *shApc/Kras*‐mt line was created by crossing in an additional LSL‐*Kras^G12D^* allele. (B) Representative IF staining for VIM, POSTN, and FN1 in *shApc/Kras^wt^* (*n* = 3) and *shApc/Kras^G12D^* (*n* = 3) GEMMs. Scale bar corresponds to 100 μm for the left most panels and 50 μm on the others. (C) Levels of VIM, POSTN, and FN1 expression on IF (*n* = 3 *shApc/Kras^wt^* and *n* = 3 *shApc/Kras^G12D^*). Median value of 3–5 quantified tumor areas per sample (density/mm2) is plotted. Error bars denote the standard error of mean. *P*‐values were computed using one‐sided Wilcoxon rank‐sum tests. (D) ECM‐related genes are consistently downregulated in KRAS‐mt across the LARC‐TIMING cohort (*n* = 42 KRAS‐wt, *n* = 34 KRAS‐mt), LARC‐TCGA cohort (*n* = 45 KRAS‐wt, *n* = 26 KRAS‐mt), and our GEMMs (*n* = 3 KRAS‐wt, *n* = 3 KRAS‐mt), while exhibiting opposing trends in KRAS‐mt (*KRAS^G12V^*) Caco2 cells (*n* = 3 KRAS‐wt, *n* = 3 KRAS‐mt) cultured in isolation. Bar plots show log_2_ fold changes in expression in KRAS‐mt vs KRAS‐wt tumors, with positive values corresponding to upregulation in KRAS‐mt tumors. *HOXB6* and *HOXB8* genes were included as reference genes based on their known upregulation in KRAS‐mt specimens. (E) GSEA enrichment plots show significant downregulation of genes in the ESTIMATE stromal gene signature for the *shApc/Kras^G12D^* mouse model. (F) No significant differences for the ESTIMATE stromal signature were observed in KRAS‐mt vs KRAS‐wt Caco2 cells.

To demonstrate that the introduction of a novel *KRAS* mutation can lead to the type of downregulation of stromal and ECM genes that we have observed in our LARC patients, we used a GEMM of CRC whereby *Apc* can be conditionally suppressed using a doxycycline‐regulated shRNA and mutant *Kras* (G12D) is activated under a Cre recombinase [28] (Fig. 3A). Consistent with our observations in human tissues, we observed lower POSTN and FN1 protein expression, but no changes in VIM in KRAS‐mt (*n* = 3) vs KRAS‐wt (*n* = 3) mouse tumors (Fig. 3B,C). ECM‐related genes that were downregulated in KRAS‐mt human tumors also exhibited lower levels of expression in tumors from KRAS‐mt mice (Fig. 3D). Furthermore, tumors from KRAS‐mt mice were associated with a diminished stromal signature based on GSEA using the ESTIMATE gene set (Fig. 3E).

### 3.6 Contribution of fibroblasts to the *KRAS*‐associated gene signature

The *KRAS*‐associated gene signature described above could be driven by intrinsic changes in the *KRAS*‐mutated epithelial cancer cells or it could be the result of changes in the levels of expression of surrounding stromal cells. To investigate this question, we examined mRNA expression levels of KRAS‐wt and KRAS‐mt Caco2 cells cultured in isolation. As the genomic background of Caco2 is KRAS‐wt [29], we created a KRAS‐mt line by inducing a *KRAS^G12V^* mutation on one allele. The differences in expression of matrisome and ECM‐related genes that we had observed in solid tumors were not replicated in KRAS‐mt vs KRAS‐wt Caco2 cells (Fig. 3D). Similarly, no significant differences were observed in terms of the stromal signature (Fig. 3F). These results suggest that the *KRAS*‐associated gene signature that we had identified in LARC reflects changes in the tumor stroma, rather than in the epithelial cancer cells.

**Fig. 4:**
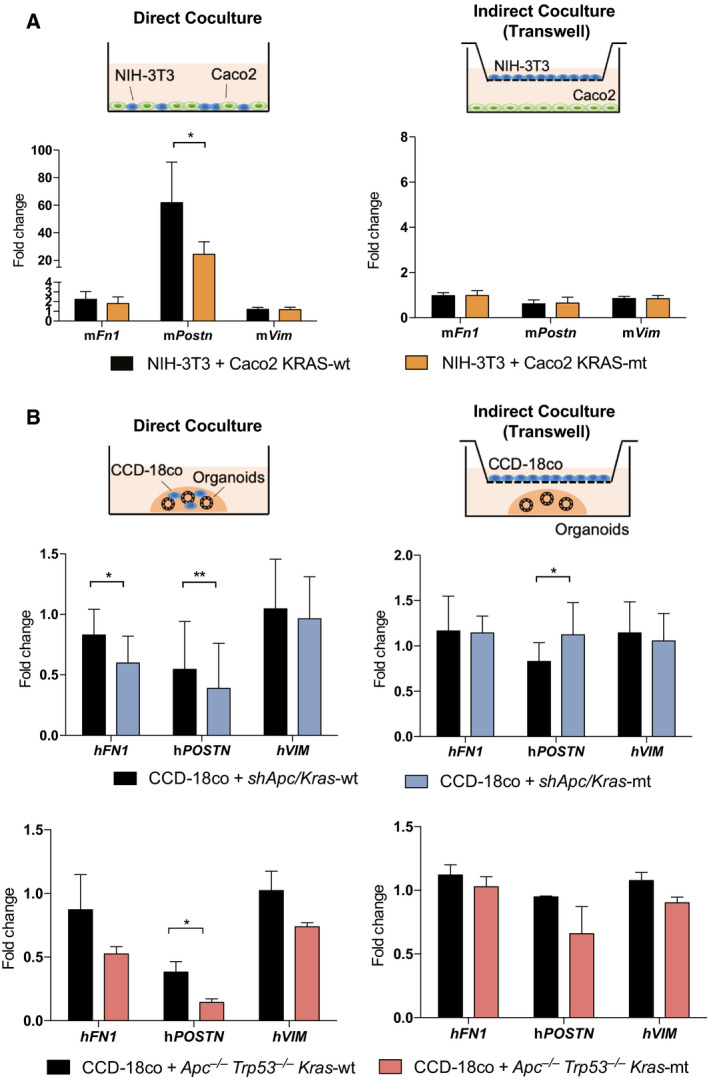
Induction of *KRAS* mutation in CRC downregulates the expression of ECM genes of the surrounding fibroblasts *in vitro*. (A) Levels of *Fn1*, *Postn*, and *Vim* mRNA from mouse fibroblasts (NIH‐3T3) in direct or indirect co‐culture with Caco2‐*KRAS^wt^* or Caco2‐*KRAS^G12V^* were assessed by RT‐PCR. (B) Levels of *FN1* and *POSTN* from human fibroblasts (CCD‐18co) grown in direct or indirect co‐culture with *shApc/Kras^wt^* or *shApc/Kras^G12D^* organoids were assessed by RT‐PCR. Additional co‐culture experiments with *Apc* ^−/−^ *Trp53* ^−/−^ *Kras^wt^* and *Apc* ^−/−^ *Trp53* ^−/−^ *Kras^G12D^* organoids were also performed. Error bars denote the standard error of mean. All experiments were done in biological and technical triplicates. One‐sided *t*‐test was performed. *, *P* < 0.05; ** *P* < 0.01.

To better understand the contribution of the stromal component to the *KRAS*‐associated gene signature, we co‐cultured mouse fibroblasts (NIH‐3T3) with human Caco2 KRAS‐mt or Caco2 KRAS‐wt tumor cells. Since the co‐culture is a mixture of mouse (fibroblast) and human (cancer cell line) cells, we used mouse‐specific RT‐PCR primers to quantify the expression of stromal genes from the fibroblasts. Expression of both mouse *Fn1* and *Postn*, but not *Vim*, was diminished in the Caco2 KRAS‐mt co‐culture compared to the Caco2 KRAS‐wt co‐culture (Fig. 4A). The reduction in mouse *Fn1* and *Postn* expression was not observed when the mouse fibroblasts and the Caco2 cells were co‐cultured physically separated by a microporous transwell membrane. These experiments suggest that the *KRAS*‐associated changes in gene expression that we have described above require the presence of fibroblasts and that these fibroblasts need to be in close proximity with epithelial cancer cells.

To further ensure the reproducibility of our results, we replicated this experiment in a 3‐dimensional culture setting using colonic organoids isolated from genetically engineered mice. KRAS‐wt (*shApc/Kras^wt^*) and KRAS‐mt (*shApc/Kras^G12D^*) mouse organoids were cultured with the human fibroblast cell line CCD‐18co in direct co‐culture or indirect transwell setting (Fig. 4B). We also employed a different set of KRAS‐wt and KRAS‐mt mouse organoids (*Apc* ^−/−^ *Trp53* ^−/−^ *Kras^wt^* and *Apc* ^−/−^ *Trp53* ^−/−^ *Kras^G12D^*) as these co‐occurring mutations are predominant in colorectal cancer and thought to be more biologically relevant. Quantification of the stromal genes expressed from the human fibroblasts was done with human‐specific RT‐PCR primers. We found reduced expression of the stromal genes *FN1* and *POSTN* in the human fibroblasts co‐cultured with KRAS‐mt compared to KRAS‐wt organoids (Fig. 4B). The difference in *FN1* and *POSTN* gene expression was not observed when the organoids and fibroblasts were co‐cultured in a transwell setting. Therefore, our data are consistent with a model where KRAS‐mt rectal cancer modulates the gene expression of surrounding fibroblasts in the microenvironment.

## 4 Discussion

In this study, we showed that KRAS‐mt LARC tumors are associated with worse response rates across all treatment groups and CMS clusters, highlighting the profound biological effects driven by *KRAS*. We found that KRAS‐mt tumors exhibited diminished expression of ECM‐related genes compared to KRAS‐wt tumors. We used GEMMs to show that the introduction of a *KRAS* mutation in the CRC cells altered the expression of ECM genes from fibroblasts in a manner that was consistent with the patterns that we had observed in human tumors. Our results using IF, as well as DNA‐based estimates of tumor purity, were consistent with a change in the transcriptional program of fibroblasts, rather than a cellularity effect. We also analyzed the subtypes of stromal cells and discovered that the gene expression levels of stromal fibroblasts and myofibroblasts were diminished in KRAS‐mt tumors, suggesting that oncogenic *KRAS* affects a diverse group of fibroblasts and not just a specific subtype.

*RAS*‐mediated modulation of the ECM in CRC has clinical and translational relevance because remodeling of the ECM is thought to play important roles in tumor growth, invasion, and treatment resistance [39, 40]. The CMS3 subgroup—which is enriched in KRAS‐mt tumors—has been shown to exhibit diminished transcriptomic signatures of stromal infiltration [21]. In this respect, our finding that a *KRAS* mutation is associated with reduced expression of ECM proteins is consistent with the CMS classification. However, not all tumors classified as CMS3 are KRAS‐mt, which suggests that other mechanisms may also contribute to the modulation of the ECM in CRC.

The effects of *KRAS* signaling in the tumor microenvironment of solid tumors have been extensively studied [41], but the role of *KRAS* in modulating the ECM remains poorly understood. The conditioned medium from a *KRAS^G12V^* transformed colon epithelial cell line has been shown to play a role in modulating the migration but not the expressive state of myofibroblasts [42]. Similarly, in our indirect co‐culture experiments, we did not observe changes in the gene expression of the fibroblasts. However, our direct co‐culture experiments showed that KRAS‐mt epithelial cancer cells downregulated the expression of ECM genes in the surrounding fibroblasts, suggesting that physical proximity of the two cells may be necessary for this mechanism. Contrary to the work in colorectal cancer, studies using pancreatic adenocarcinoma models have found that *KRAS^G12D^* mutations activate stromal cells and upregulate multiple ECM proteins [43]. These findings suggest that the mechanisms behind *KRAS* modulation of the ECM may be cancer‐type specific.

The unique set of human rectal cancer specimens that we have analyzed provides an anatomically homogenous population in which to study both cancer cell‐intrinsic and stromal changes driven by *KRAS* mutations. Gene expression microarrays were used for transcriptional profiling of patients in our main cohort because this was the most prevalent sequencing platform at the time when specimens from the TIMING trial were originally collected and processed; however, we have shown that our results can be reliably reproduced using alternative approaches such as RT‐PCR and RNA‐Seq. Genomic profiling of *KRAS* mutational status was done using Sanger sequencing in a large subset of our cases, and therefore, we cannot exclude the possibility that other important members of the *RAS* signaling pathway such as *NRAS* or *BRAF* were mutated in a small fraction of our KRAS‐wt samples. While our patient cohort presented a diverse catalogue of *KRAS* mutations, we selected *KRAS^G12D^* and *KRAS^G12V^* to study KRAS‐mt tumors in our experimental models. This might limit the generalizability of these results as different *KRAS* mutations have been hypothesized to have diverse tumorigenic effects [12, 44]. *TP53* and *KRAS* double mutant tumors have been reported to exhibit particularly low rates of response to CRT in LARC [12], but we have not analyzed them as a separate entity in this study due to the small sample size. Finally, a number of previous studies have shown that *KRAS* mutant cancer cells have immunoregulatory effects that extend beyond the ECM changes reported in our study to affect other elements of the tumor microenvironment [41]. Future analyses combining single‐cell sequencing technologies and multiplex immunofluorescence will make it possible to better investigate this clinically relevant question.

## 5 Conclusions

In conclusion, we present the first transcriptomic analysis of samples accrued through the TIMING trial for patients with LARC. Our results show pronounced remodeling of the ECM in KRAS‐mt tumors and altered transcriptional programs in their surrounding fibroblasts mediated by *KRAS*. While more work is clearly needed to better understand how this contributes to the malignant potential of KRAS‐mt rectal cancer, our analyses constitute a step forward in the characterization of the dysregulated biological processes orchestrated by *KRAS*.

## Conflict of interest

JGA has received honorarium for being a consultant with the following: Medtronics, Ethicon J&J, Da Vinci Intuitive Surgical. JJS has received travel support from Intuitive Surgical Inc. for fellow education (2015) and has served as a clinical advisor for Guardant Health, Inc (2019). The other co‐authors have no conflicts of interest to disclose.

## Author contributions

JKK involved in conceptualization, formal analysis, writing—original draft, visualization, and project administration; MRC contributed to investigation, writing—original draft and visualization; SHC contributed to methodology, validation, formal analysis, and investigation; XQ contributed to software, validation, data curation, and visualization; CTC methodology and validation; ME made investigation; LF contributed data curation; OC made investigation; FMB, LED, KO, and KMT contributed to methodology; BS, DY, and SF made investigation; PBP and CL made resources; JS made supervision; JJS and SL contributed to conceptualization, writing‐review/editing, and supervision; RP contributed conceptualization, software, validation, formal analysis, data curation, and writing‐original draft; FSV contributed conceptualization, software, validation, formal analysis, writing‐original draft, visualization, and project administration; and JGA involved in conceptualization, resources, writing‐review/editing, and funding acquisition.

### Peer Review

The peer review history for this article is available at https://publons.com/publon/10.1002/1878‐0261.12960.

## Supporting information

**Fig. S1**. CMS clustering of samples in the LARC‐TIMING cohort. Heatmap shows varying levels of gene expression across samples stratified by consensus molecular subtype (CMS). Within each CMS subtype, samples were further grouped according to *KRAS* mutational status. Genes were grouped vertically into relevant sets using a previously published set of gene expression signatures of biological relevance in colorectal cancer.

**Fig. S2**. Validation of KRAS‐mt transcriptional signature using tumors from the LARC‐TCGA and the CRC‐RT‐PCR cohorts. A: RT‐PCR validation of selected differentially expressed genes in the CRC‐RT‐PCR cohort (*n* = 30 KRAS‐wt, *n* = 15 KRAS‐mt). Experiments were done in technical triplicates; three independent experiments were performed. Fold change was calculated after normalization to human 18s rRNA using the $2^{‐\Delta \Delta C_{t}}$ method. Error bars show standard error of the mean. a one‐sided Wilcoxon rank‐sum test was performed. *P* < 0.05 is denoted by asterisk. B: External validation of upregulated and downregulated gene sets using the LARC‐TCGA cohort (*n* = 45 KRAS‐wt, *n* = 26 KRAS‐mt). The volcano plot is used to visualize concordance between genes that were upregulated and downregulated in KRAS‐mt vs KRAS‐wt across the two cohorts. C: Validation of the top 15 pathways using the LARC‐TCGA cohort. D: Validation of the stromal signature using the LARC‐TCGA cohort. *P*‐values were computed using gene set enrichment analysis (GSEA). E: Tumor purity estimates of KRAS‐wt (*n* = 45) and KRAS‐mt (*n* = 26) samples in the LARC‐TCGA cohort, computed using the ABSOLUTE algorithm (*P* = 0.38). Box whisker plot is shown; whiskers represent 1.5× the interquartile range. *P*‐value is computed by one‐sided Wilcoxon rank‐sum test.

**Fig. S3**. Analysis of VIM, POSTN, and FN1 protein expression in normal colon. A: Representative images of IF staining in normal human colon. Scale bars correspond to 2mm on the left panels and 50 μm on the right panels. B: Representative images of IF staining in normal mouse colon. Scale bars correspond to 200 μm on the left panels and 50 μm on the right panels.

**Fig. S4**. Analysis of stromal subpopulations in KRAS‐mt vs KRAS‐wt LARC‐TIMING patients. A: Heatmap showing hierarchical clustering of patients and stromal subtypes using single‐sample gene set enrichment analysis (ssGSEA) scores for the set of signatures from Lee et al. Patients (*n* = 76) were initially stratified according to *KRAS* status. B: Gene set enrichment analysis (GSEA) results comparing KRAS‐mt (*n* = 34) vs KRAS‐wt (*n* = 42) tumors. Stromal subtypes that were significantly enriched in KRAS‐wt samples are highlighted in light blue, while subtypes enriched in KRAS‐mt tumors are highlighted in pale red.

**Table S1**. Clinicopathological Features of LARC‐TIMING Patients.
**Table S2**. List of LARC‐TCGA Barcodes.
**Table S3**. Differentially Expressed Genes in KRAS‐mt vs KRAS‐wt Patients from the LARC‐TIMING Cohort.
**Table S4**. Differentially Expressed Genes in KRAS‐mt vs KRAS‐wt Patients from the LARC‐TCGA cohort.
**Table S5**. GSEA Results from Comparing KRAS‐mt vs KRAS‐wt Patients in the LARC‐TIMING Cohort.
**Table S6**. GSEA Results from Comparing KRAS‐mt vs KRAS‐wt Patients in the LARC‐TCGA Cohort.
**Table S7**. Primers Used for RT‐PCR Analyses.
**Table S8**. List of Antibodies Used for IHC.

## Data Availability

The gene expression data for the LARC‐TIMING cohort are uploaded in GEO (GSE170999, https://www.ncbi.nlm.nih.gov/geo/query/acc.cgi?acc=GSE170999).
