# Supplementary figures and images for: KRAS mutant rectal cancer cells interact with surrounding fibroblasts to deplete the extracellular matrix
Source: Mol Oncol. 2021 Jun 15;15(10):2766–81. doi: 10.1002/1878-0261.12960 (PMC8486594; doi:10.1002/1878-0261.12960)

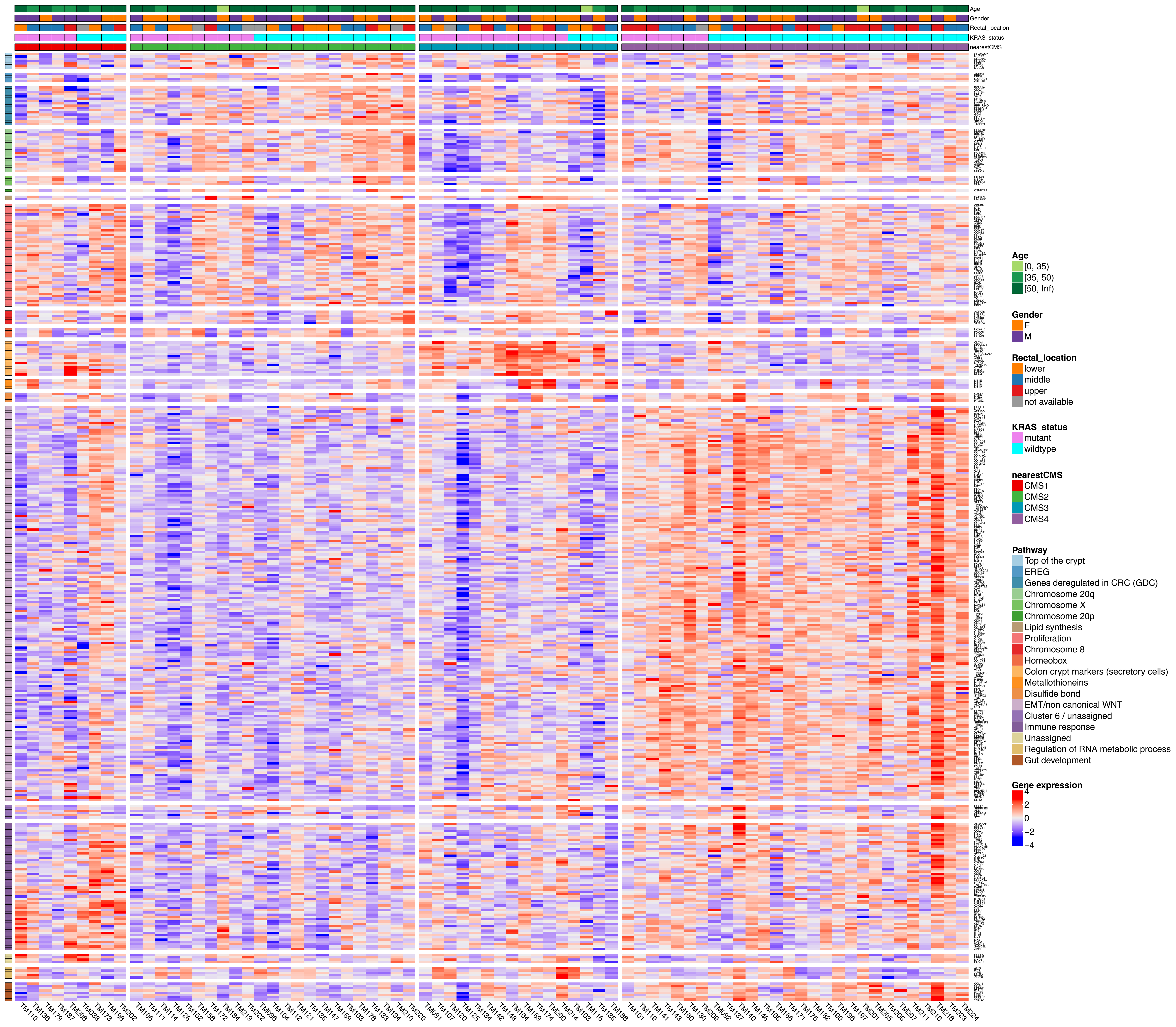

Supplement: Supplementary file 1 — Fig. S1. CMS clustering of samples in the LARC‐TIMING cohort. Heatmap shows varying levels of gene expression across samples stratified by consensus molecular subtype (CMS). Within each CMS subtype, samples were further grouped according to KRAS mutational status. Genes were grouped vertically into relevant sets using a previously published set of gene expression signatures of biological relevance in colorectal cancer. [file MOL2-15-2766-s005.pdf]

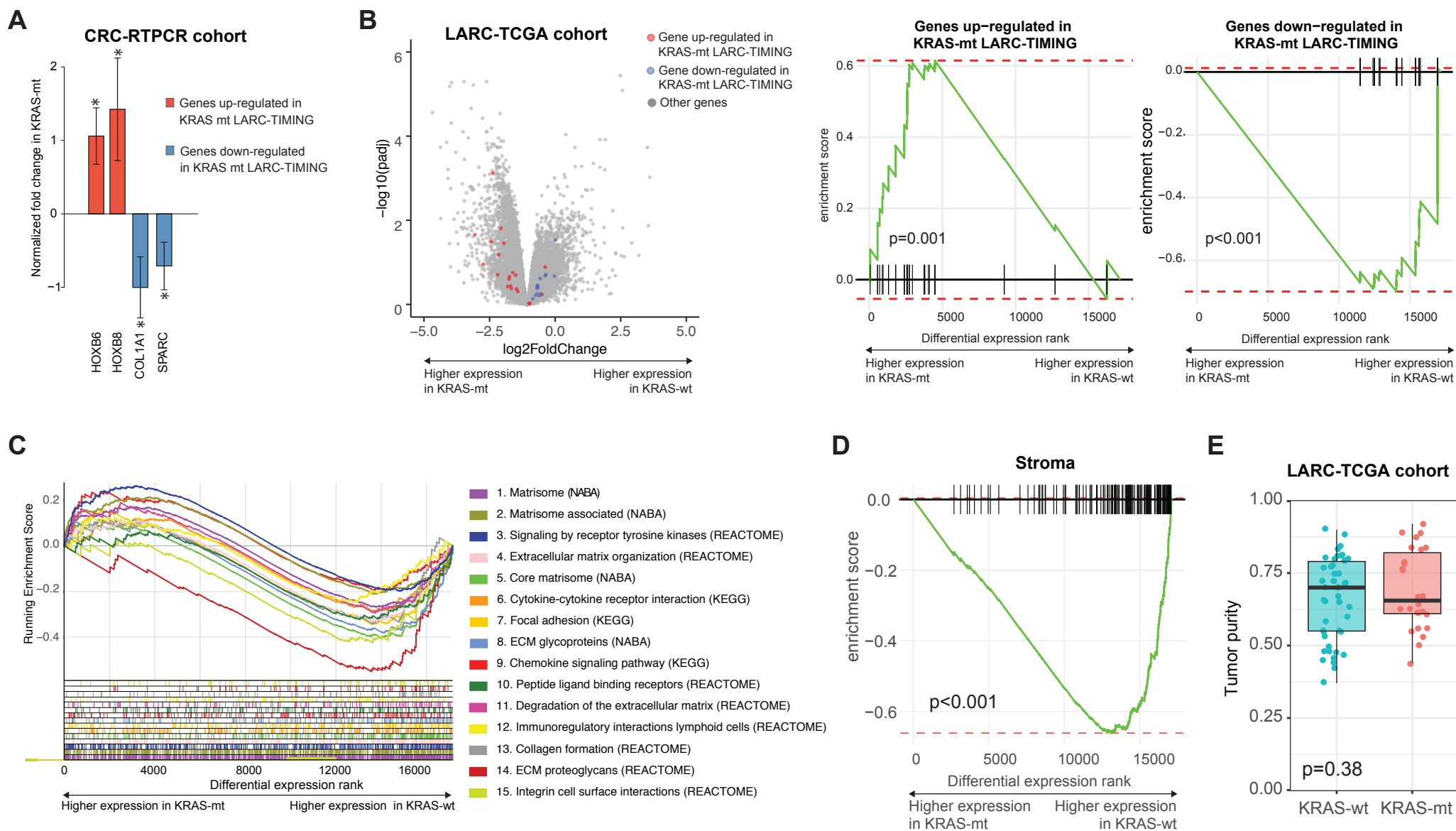

Figure S2

Supplement: Supplementary file 2 — Fig. S2. Validation of KRAS‐mt transcriptional signature using tumors from the LARC‐TCGA and the CRC‐RT‐PCR cohorts. A: RT‐PCR validation of selected differentially expressed genes in the CRC‐RT‐PCR cohort (n = 30 KRAS‐wt, n = 15 KRAS‐mt). Experiments were done in technical triplicates; three independent experiments were performed. Fold change was calculated after normalization to human 18s rRNA using the 2‐ΔΔCt method. Error bars show standard error of the mean. a one‐sided Wilcoxon rank‐sum test was performed. P < 0.05 is denoted by asterisk. B: External validation of upregulated and downregulated gene sets using the LARC‐TCGA cohort (n = 45 KRAS‐wt, n = 26 KRAS‐mt). The volcano plot is used to visualize concordance between genes that were upregulated and downregulated in KRAS‐mt vs KRAS‐wt across the two cohorts. C: Validation of the top 15 pathways using the LARC‐TCGA cohort. D: Validation of the stromal signature using the LARC‐TCGA cohort. P‐values were computed using gene set enrichment analysis (GSEA). E: Tumor purity estimates of KRAS‐wt (n = 45) and KRAS‐mt (n = 26) samples in the LARC‐TCGA cohort, computed using the ABSOLUTE algorithm (P = 0.38). Box whisker plot is shown; whiskers represent 1.5× the interquartile range. P‐value is computed by one‐sided Wilcoxon rank‐sum test. [file MOL2-15-2766-s001.pdf]

**A**

Human normal colon tissue staining

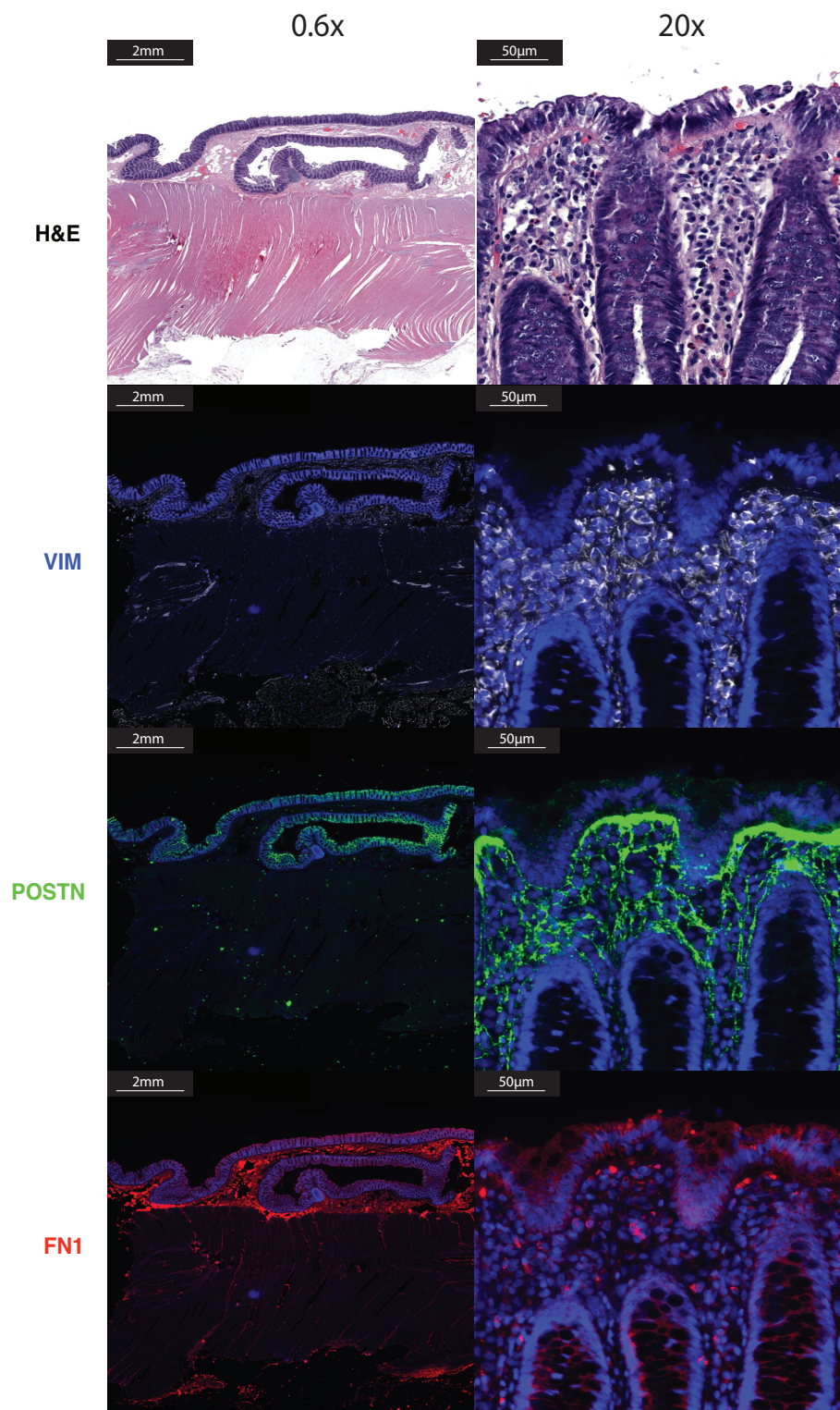**B**

Mouse normal colon tissue staining

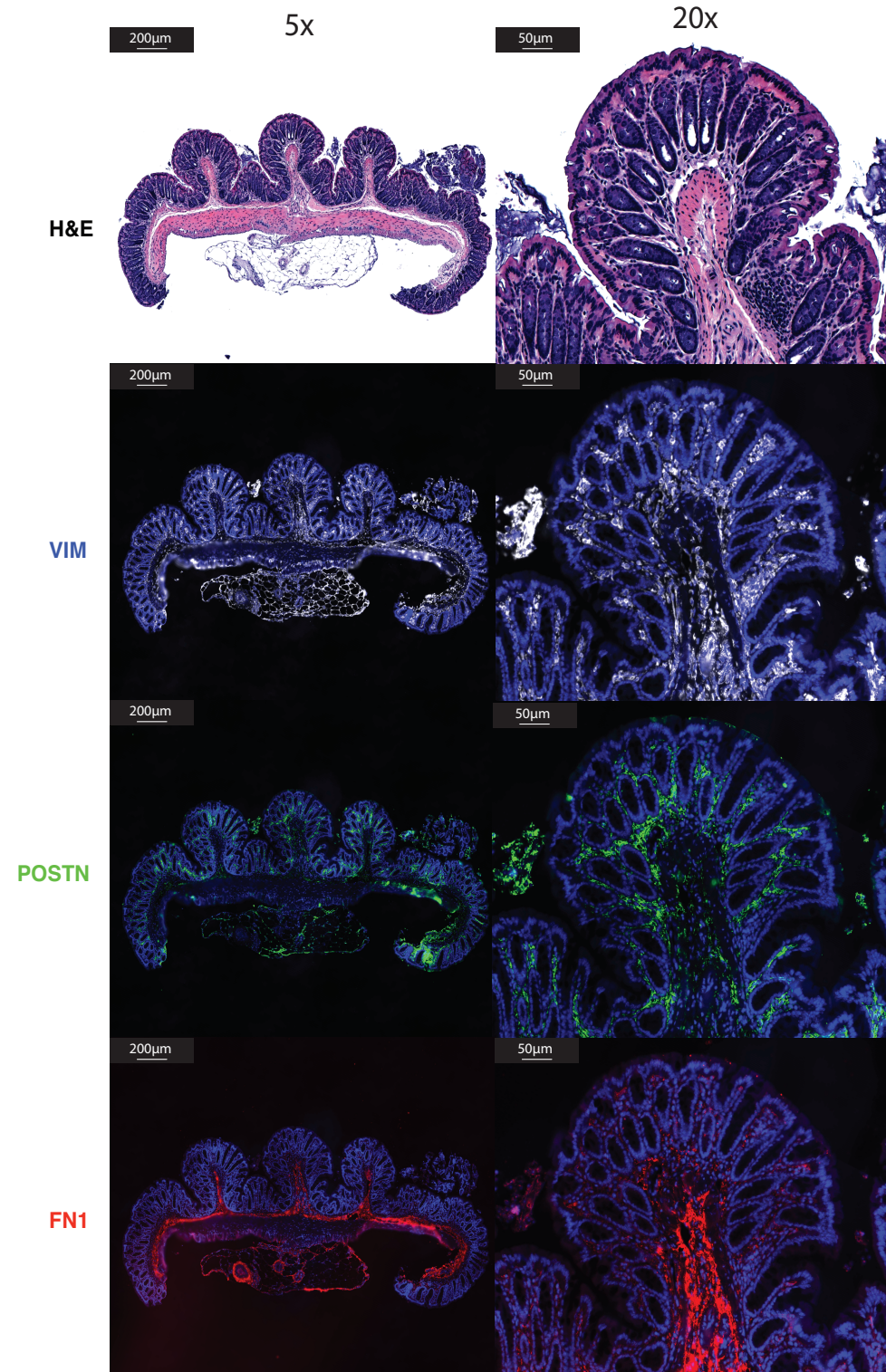

Figure S3

Supplement: Supplementary file 3 — Fig. S3. Analysis of VIM, POSTN, and FN1 protein expression in normal colon. A: Representative images of IF staining in normal human colon. Scale bars correspond to 2mm on the left panels and 50 μm on the right panels. B: Representative images of IF staining in normal mouse colon. Scale bars correspond to 200 μm on the left panels and 50 μm on the right panels. [file MOL2-15-2766-s004.pdf]

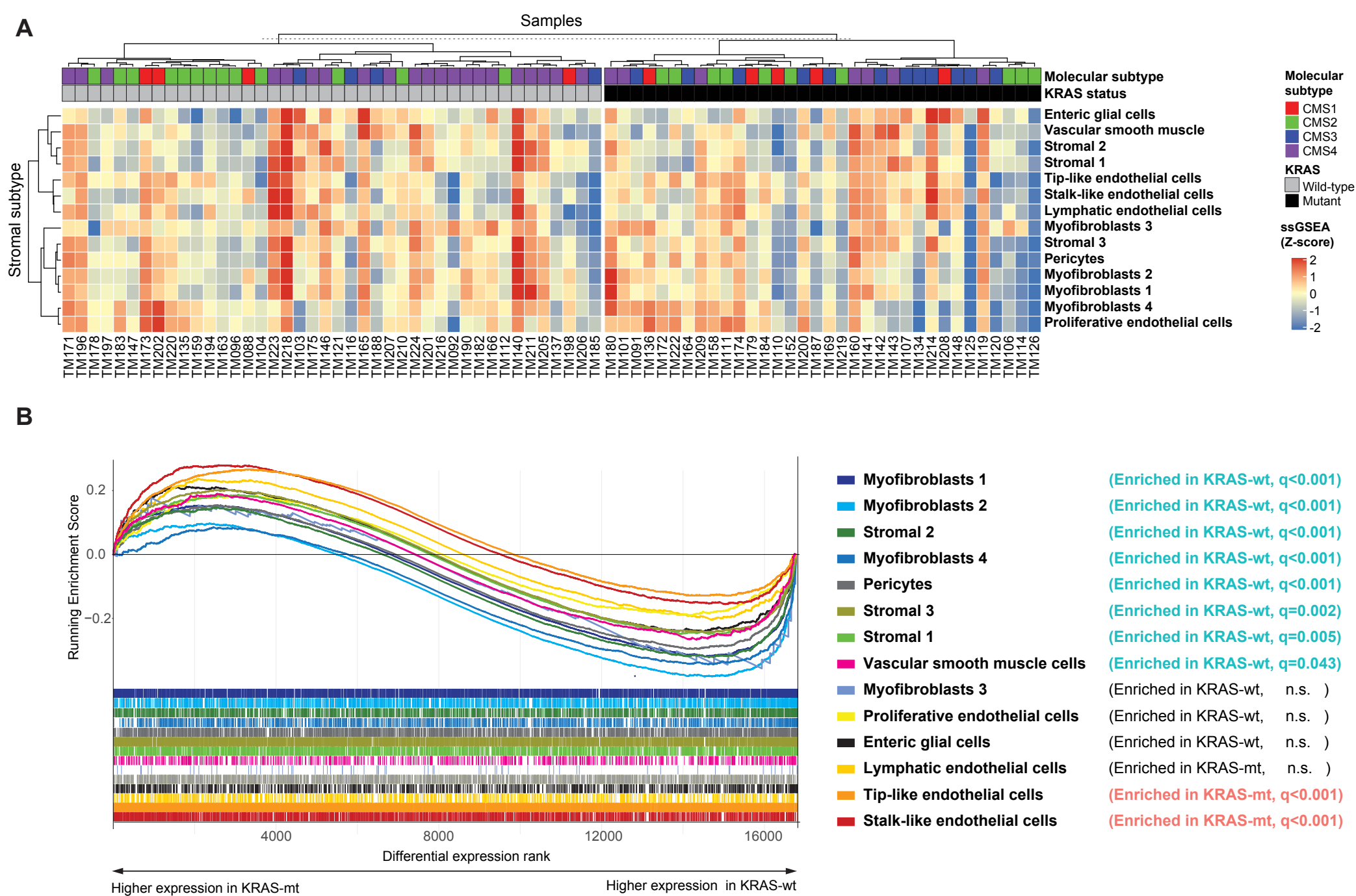

Figure S4

Supplement: Supplementary file 4 — Fig. S4. Analysis of stromal subpopulations in KRAS‐mt vs KRAS‐wt LARC‐TIMING patients. A: Heatmap showing hierarchical clustering of patients and stromal subtypes using single‐sample gene set enrichment analysis (ssGSEA) scores for the set of signatures from Lee et al. Patients (n = 76) were initially stratified according to KRAS status. B: Gene set enrichment analysis (GSEA) results comparing KRAS‐mt (n = 34) vs KRAS‐wt (n = 42) tumors. Stromal subtypes that were significantly enriched in KRAS‐wt samples are highlighted in light blue, while subtypes enriched in KRAS‐mt tumors are highlighted in pale red. [file MOL2-15-2766-s003.pdf]
